# The association between glycemic index and glycemic load and quality of life among overweight and obese women: a cross-sectional study

**DOI:** 10.1186/s40795-022-00668-8

**Published:** 2023-02-13

**Authors:** Niloufar Rasaei, Melika Fallah, Fatemeh Gholami, Mehdi Karimi, Sahar Noori, Niki Bahrampour, Cain C. T. Clark, Khadijeh Mirzaei

**Affiliations:** 1 Department of Community Nutrition, School of Nutritional Sciences and Dietetics, Tehran University of Medical Sciences(TUMS), P.O. Box, 14155-6117 Tehran, Iran; 2 Department of Nutrition, Science and Research Branch, and Islamic Azad University, Tehran, Iran; 3 Centre for Intelligent Healthcare, Coventry University, Coventry, CV1 5FB UK; 4 Food Microbiology Research Center, Tehran University of Medical Sciences, Tehran, Iran

**Keywords:** Quality of life, Glycemic index, Glycemic load, Diet, Overweight, Obesity

## Abstract

**Background:**

The association between different dietary approaches and quality of life (QoL) has been well-demonstrated in previous research. However, the relationship between glycemic index (GI) and glycemic load (GL) with different dimensions of QoL has not been established. Therefore, we aimed to investigate the relationship between GI and GL with QoL in overweight and obese women.

**Methods:**

Two hundred seventy-six overweight and obese women (body mass index (BMI) ≥ 30 kg/m^2^), aged 18–64 years old, were included in this cross-sectional study. The amount of dietary intake and GI and GL indexes were established using a valid and reliable Food Frequency Questionnaire (FFQ) containing 147 items. Body composition (using bioimpedance analysis), anthropometrics, and physical activity were assessed. Insulin resistance (HOMA-IR) and hs-CRP were also measured, whilst QoL was measured using the SF-36 (short-form-36), self-administered, questionnaire.

**Result:**

Analyses were performed using multivariable linear regression, considering a wide range of confounding variables, such as age, physical activity, BMI, education, job, smoking, and marriage. We found a significant negative association between glycemic load and quality of life (β = -0.07, 95%CI = -0.13_ -0.01, *p* = 0.01). No significant associations were observed between glycemic index and quality of life (β = -0.03, 95%CI = -0.81_ 0.75, *p* = 0.93).

**Conclusion:**

We observed a significant negative association between QoL and GL, but not GI, among overweight and obese women in Iran. Our results need to be confirmed with further well-designed and adequately powered studies that control for clinical confounders.

## Introduction

Over the past decade, the evaluation of quality of life)QoL( has become an essential clinical and research outcome measurement [1]. QoL is often used as a comprehensive concept, and when used in health care, it refers primarily to the physical components and occasionally extends to psychological components [2]. The World Health Organization (WHO) defines quality of life as an individual's perception of his or her position in life, within the culture and value system in which he or she lives, and his or her goals, expectations, patterns, and concerns [3]. QoL includes [1] physical aspects, such as pain, fatigue, energy, sleep and rest, [2] psychological aspects, such as self-esteem, memory, positive and negative emotions, and perception of body image and appearance, [3] Social aspects that focus primarily on personal relationships; and [4] Environmental aspects such as security, finance, leisure and information [3]. Diet is one of several environmental factors which can directly affect a person's QoL [4–8], and one of the most important components in the prediction of health outcomes is glycemic index and glycemic load of a diet [9].

Foods containing carbohydrate have a wide range of effects on Glycemic response (GR) [10]. The glycemic index (GI) estimates the rate at which carbohydrates are broken down during digestion and the rate at which they are absorbed into the bloodstream [11]. Several factors determine the GI of a food, including the type of carbohydrate, protein content, fat, pH, amount and type of fiber, and finally the particle size of the food [12]. Carbohydrate-rich foods that break down quickly and absorbed into the bloodstream are classified as high-GI foods, which leads to a rapid rise in blood glucose and an insulin response. Conversely, foods with a low GI have a slower and lower effect on postprandial blood glucose and insulin response level, respectively [13]. Given that the glycemic index does not provide information on how increased and prolonged of glycemia when consuming a certain amount of a carbohydrate-rich food, a separate measure called the glycemic load (GL) does both, therein providing a more accurate picture of a food’s real-life impact on postprandial glycemia [13]. The term GL combines the GI of a food or diet with the amount of carbohydrates in a given amount of a food, meal, or diet [14].

Various studies have examined the association of different types of diets, e.g. Mediterranean diet (Med Diet) [5], low carbohydrate diet (LCD) [6], therapeutic lifestyle changes diet [7], pulse-based diet [7], and fasting mimicking diet [8], with quality of life in different populations, indicating a positive association between adherence to these diets and several dimensions of QoL. In a previous cross-sectional study the association between adherence to a traditional Med Diet and health-related QoL (HRQoL) was investigated in older Spanish women and men with overweight or obesity harboring the metabolic syndrome. Participants aged 55–70 years and 6430 women and men were included in the study. HRQoL was assesed with 36-item questionnaire and adherence to Med Diet was assessed with 17-item questionnaire. Higher adherence to the Med Diet had a positive relation with several dimensions of HRQoL [5]. A prospective, randomized trial study on 61 obese adults with Type 2 diabetes (body mass index (BMI): 32.7 ± 5.4 kg/m^2^) was designed to compare the effects of a 2-year intervention with a LCD or low-fat diet (LFD) on HRQoL. LFD included 55–60 energy percent (E%) and LCD included 20 E% from carbohydrates. The Short Form-36 (SF-36) questionnaire was used to measure HRQoL in this clinical practice. After one year of treatment with LCD, improvements in HRQoL occurred [15]. Although various studies with different results have been published on the different types of diets and QoL [4–7], to date, no study has investigated the association between GI and GL with QoL. Therefore, for the first time in Iran, we examined the associations between GI and GL with QoL in overweight and obese women.

## Methods

### Study population

**Fig. 1:**
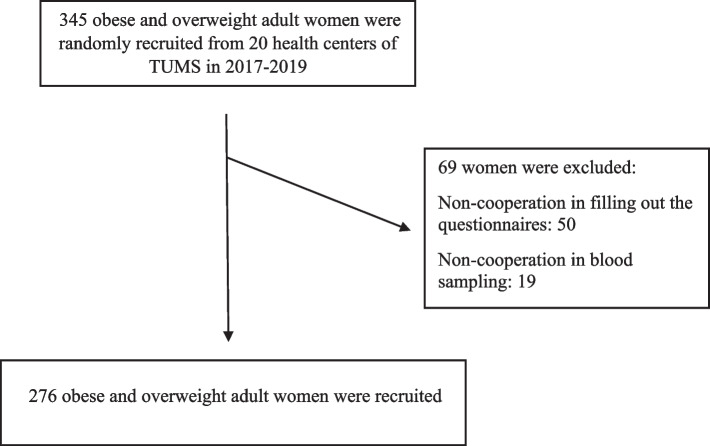
The flow chart of study design

This cross-sectional study was conducted on 276 obese and overweight adult women, recruited from health care centers of Tehran city, Iran, between 2017–2019. Multi-stage simple random sampling was used. From all health centers of the Tehran University of medical sciences (TUMS), 20 health centers randomly were selected. Sampling was such that if women who were referred to Tehran health centers, met the inclusion criteria, were selected randomly to enter the study. Finally 276 women were recruited. The inclusion criteria were: being 18 to 64 years of age and having a BMI ≥ 30 kg/m^2^. The exclusion criteria were: cardiovascular disease, diabetes type 1 and 2, kidney disease, thyroid disease, malignancies, menopause, pregnancy, lactation, smoking, any acute or chronic diseases, consuming weight loss supplements, following a weight-loss diet over the past year, receiving lipid, glucose and blood pressure lowering drugs. Each participant was informed completely regarding the study protocol, the objectives of the study were explained to them, and finally written consent was obtained from all participants. Privacy and confidentiality were maintained. This study was conducted according to the ethical standards of the Human Research Ethics Committee of the Tehran University of Medical Sciences (IR.TUMS.MEDICINE.REC.1399.636), and in concordance with the Declaration of Helsinki (Fig. 1).

### Biochemical assessments

Twelve cc fasting venous blood samples were drawn from participants, following a 10–12 h fast, between 8: 00 and 10: 00 a.m. The blood samples were immediately centrifuged, aliquoted, and stored at –80 °C, and were analyzed by using a single assay technique. Measuring serum fasting blood glucose (FBS) with a colorimetric method was used from glucose oxidase–phenol 4-aminoantipyrine peroxidase (GOD-PAP). Triglyceride (TG) and Total cholesterol (TC) were measured by glycerol-3-phosphate oxidase–phenol 4-aminoantipyrine peroxidase (GPOPAP), enzymatic endpoint, and Low-density-lipoprotein (LDL) and high-density lipoprotein (HDL) cholesterol measured by direct enzymatic clearance assay. Serum high-sensitive C-reactive protein (hs-CRP) was evaluated with the use of the immunoturbidimetric assay. IR was estimated by homeostasis model assessment (HOMA), which was calculated according to the following equation: HOMA = [Fasting Plasma Glucose (mmol/L) × Fasting Plasma Insulin (mIU/L)]/22.5 [16]. All detections were performed using Randox Laboratories kit (Hitachi 902).

### Body composition analysis

The body composition was assessed with a bioelectrical impedance analyzer (BIA) InBody 770 scanner (Inbody Co., Seoul, Korea) between 8–9 am after 12 h of overnight fasting that strictly following the procedure, techniques and precaution of the manufacturer’s protocol [17]. Based on the manufacturer’s instructions, all of participants were asked to remove extra clothes, including coat, sweater, shoes, and remove metal utensils/jewelry, such as rings, watches, and clothes. The examination takes nearly 20 s, and the BIA calculates waist circumference (WC), skeletal muscle mass (SMM), waist to hip ratio (WHR), fat free mass (FFM), and fat mass (FM).

### Anthropometric measures

Height was measured by a Seca scale in standing position beside the wall, while barefoot and shoulders touching the wall, to the nearest 0.5 cm [18]. Hip circumference (HC) was measured to the nearest 0.5 cm, using a non-stretch tape measure. The weight and BMI were measured by BIA.

### Physical activity assessment

Physical activity (PA) was appraised using the short-form of the International Physical Activity Questionnaire (IPAQ). This questionnaire calculates the PA of all participants during the past 7 days. The validity and reliability of IPAQ questionnaires has been confirmed across 12 countries. The criterion reliability of this questionnaires had a Spearman’s ρ of around 0.8. The median ρ for the validity has been reported around 0.30, which was similar to other validation studies. IPAQ is a validated self-reported seven-item measure of physical activity that indicates PA (vigorous, moderate, walking, and inactive) over the last week, and then, according to guidelines, the values were multiplied by their metabolic equivalent (MET) quantities and the acquired numbers were summed together to calculate MET/min/week values [19].

### Dietary intake assessment

A 147-item semi-quantitative Food Frequency Questionnaire (FFQ) was employed by a trained dietitian to assess the usual dietary intake of the participants. The validity and reliability of the FFQ have been previously reported [20]. Participants reported their frequency of consumption of a given serving of each food item during the previous year on a daily, weekly, monthly, or yearly basis. Portion sizes of the consumed foods were converted to grams and milliliters using household measurements [21] and then individuals’ dietary intake data were analyzed using the *Nutritionist* IV software.

### Glycemic index and glycemic load calculation

Subjects in the present study were tested during 3–5 separated occasions in the morning, after they had fasted overnight. On 2 occasions, the subjects ate test meals comprising one of the test foods—the portion size of each test food contained 50 g available carbohydrate. The test meal, on the other occasions, included the reference food, which could be 50 g glucose, 55 g dextrose, or 50 g available carbohydrate from white bread. After a fasting blood sample was drawn on every occasion, the subjects consumed the test meal. Further blood samples were taken at 15, 30, 45, 60, 90, and 120 min after they began to eat. After each food test for each participant based on the reference food in the same subject, the area under the GR curve (AUC) was demonstrated as a percentage of the mean AUC. To calculate the GI of food for all participants the mean of these values was used. To convert white bread as a reference food to a glucose scale, we multiplied the GI values by 0.71 (i.e. the GI of glucose = 100) [22]. Using the following formula, the total dietary GI was calculated: ∑ (GIa X available carbohydratea)/total available carbohydrate.

**Fig. 2:**
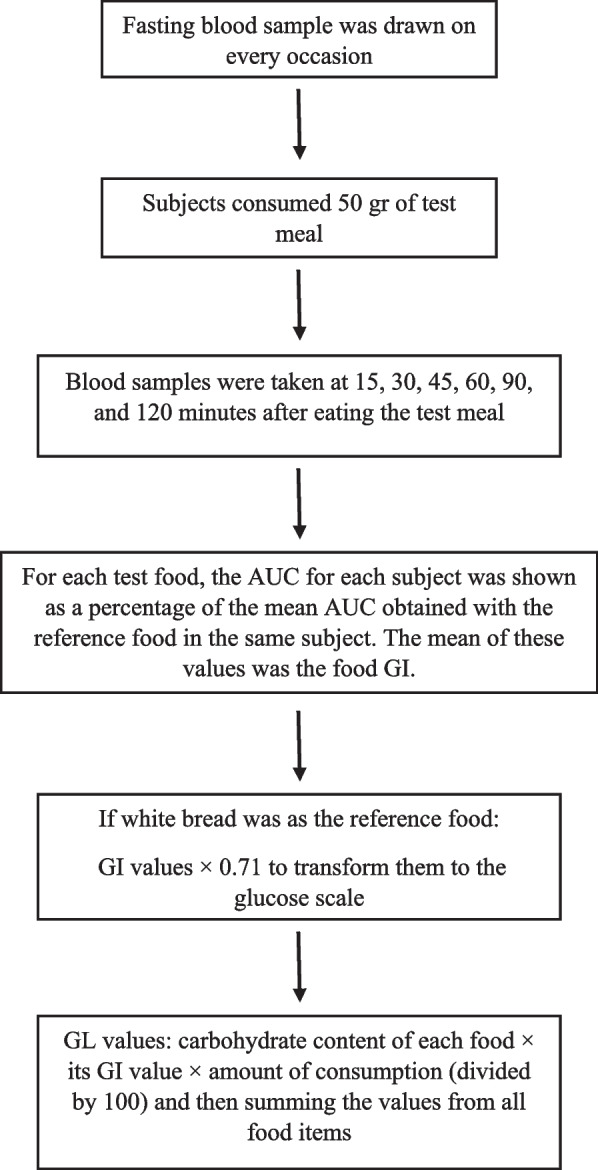
Glycemic Index and glycemic load calculation

To calculate available carbohydrates, fiber was reduced from total carbohydrates, which were derived from the USDA Department of Foodstuffs Chart [23]. To calculate the dietary GL we used the following formula: (total glycemic index * total carbohydrate available) / 100 (Fig. 2).

### Quality of life assessment

The SF-36 is a short-form, self-administered, quality of life scoring questionnaire that consists of 36 questions, 35 of which are compressed into eight multi-item scales including: physical functioning (PF), role-physical (RP), bodily pain (BP), general health (GH), vitality (VT), social functioning (SF), role emotional (RE), and mental health (MH). (1) PF is a 10-question scale that captures abilities to deal with the physical requirement of life, such as attending to personal needs, walking, and flexibility. (2) RP is a four-item scale that evaluates the extent to which physical capabilities limit activity. (3) BP is a two-item scale that evaluates the perceived amount of pain experienced during the most recent 4 weeks and the extent to which that pain interfered with normal work activities. (4) GH is a five-item scale that evaluates general health in terms of personal perception. (5) VT is a four-item scale that evaluates feeling of pep, energy, and fatigue. (6) SF is a two-item scale that evaluates the extent and amount of time, if any, that physical health or emotional problems interfered with family, friends, and other social interactions during the most recent 4 weeks. (7) RE is a three-item scale that evaluates the extent, if any, to which emotional factors interfere with work or other activities. (8) MH is a five-item scale that evaluates feelings principally of anxiety and depression [24, 25]. The SF also includes a question self-evaluating health changes in the past year (reported health), which does not belong to the eight dimensions, or the total SF-36 score. Each of these 8 dimensions has a score between 0 (worst health) to 100 (best health) [26–28].

### Statistical analysis

Statistical analyses were done using SPSS software (version 23, SPSS Inc., Chicago, IL, USA) and statistically significant was defined as *p* < 0.05. Normality of the data was checked by the Kolmogorov–Smirnov test. The distribution of categorical factors (educational status, supplement use, income, job, and marriage) across tertiles of GI and GL were performed using the Chi-square test. The comparison of the continuous variables and QoL items across across tertiles of GI and GL were investigated using analysis of variance (ANOVA) test. The analysis of covariance (ANCOVA) was applied for estimating energy-adjusted women’s dietary intakes across tertiles of GI and GL. Linear regression test was performed for assessing the associations of GI and GL with QoL among obese and overweight female subjects in three different models: crude model; model 1, adjusted for age, PA and BMI; model 2, adjusted for model 1 plus education status, job, smoking, and marri.

## Results

In total, 276 women were included in the statistical analysis. The means and standard deviation (SD) of the GL and GI in this study were 211.91 ± 76.16 and 56.71 ± 6.15, respectively. The mean (SD) of age, weight, and BMI of individuals were 36.82 ± 9.23 years, 79.74 ± 10.59 kg and 30.73 ± 3.65 kg/m^2^, respectively. The mean (SD) quality of life (SF-36 –total) score of the participants was 60.97 ± 29.42.

In this cross-sectional study, socio-economic status, such as marriage, occupation, and education were also examined. The results displayed that 161 (58.3%) participants were housekeepers and 216 (78.3%) participants were married. The majority of participants were educated to diploma (130 (47.1%)) and bachelor or higher (130 (47.1%)) level. Sixteen (5.8%) participants were smokers.

### General characteristics of participants across two groups of GI and GL

A total of 276 Iranian women were categorized based on GL and GI. Participants’ characteristics in relation to different categories of GL and GI are presented in Table 1. Also, the results displayed a significant difference across GI for total cholesterol (*p* = 0.007). No significant differences across the GL and GI with other variables were seen (Table 1).

**Table 1:** General characteristics of participants across two groups of GI and GL

**Variables** ^a^	**GI**					**GL**		
	T1	T2	T3	*P*-value	T1	T2	T3	*P*-value
**Education%(n)**								
Illiterate	33.3 (1)	66.7 (2)	0.00 (0)	0.28	66.7 (2)	33.3 (1)	0.00 (0)	0.38
Primary education	23.1 (3)	15.4 (2)	61.5 (8)		23.1 (3)	46.2 (6)	30.8 (4)	
intermediate Education	17.6 (3)	35.3 (6)	47.1 (8)		41.2 (7)	17.6 (3)	41.2 (7)	
High school education	28.6 (2)	42.9 (3)	28.6 (2)		0.00 (0)	71.4 (5)	28.6 (2)	
Diploma	30.9 (25)	29.6 (24)	39.5 (32)		28.4 (23)	34.6 (28)	37.0 (30)	
Postgraduate education	43.5 (10)	30.4 (7)	26.1(6)		39.1 (9)	34.8 (8)	26.1(6)	
Bachelor's degree and higher	36.9 (48)	36.2 (47)	26.9 (35)		36.9 (48)	30.8 (40)	32.3 (42)	
**Job%(n)**								
Housekeeper	31.7 (51)	34.8 (56)	33.5 (54)	0.66	29.2 (47)	35.4 (57)	35.4 (57)	0.51
Labor	0.0	33.3 (1)	66.7 (2)		33.3 (1)	0.00 (0)	66.7 (2)	
Management employee	31.9 (15)	34.0(16)	34 (16)		44.7 (21)	31.9 (15)	23.4 (11)	
Non-managerial employee	50.0 (18)	22.2 (8)	27.8 (10)		41.7 (15)	30.6 (11)	27.8 (10)	
household jobs	16.7 (1)	50.0 (3)	33.3 (2)		50.0 (3)	16.7 (1)	33.3 (2)	
University student	29.4 (5)	41.2 (7)	29.4 (5)		23.5 (4)	29.4 (5)	47.1 (8)	
**Marriage%(n)**								
Married	35.2 (76)	31.00 (67)	33.8 (73)	0.68	35.2 (76)	32.9 (71)	31.9 (69)	0.31
Single	30.00 (15)	42.00 (21)	28.0 (14)		32.0 (16)	32.0 (16)	36.0 (18)	
Away from spouse more than 6 month	0.0 (0)	0.0 (0)	100.0 (1)		0.0 (0)	100 (1)	0.00 (0)	
Dead spouse	0.0 (0)	50.0 (1)	50.0 (1)		0.0 (0)	0.0 (0	100.0 (2)	
Divorce	20.0 (1)	40.0 (2)	40.0 (2)		0.0 (0)	60.0(3)	40.0 (2)	
**Supplementation%(n)**								
Yes	36.9 (48)	29.2 (38)	33.8 (44)	0.82	31.5 (41)	32.3 (42)	36.2 (47)	0.42
No	37.0 (34)	32.6 (30)	30.4 (28)		38.0 (35)	33.7 (31)	28.3 (26)	
**Smoking%(n)**								
Yes	37.5 (6)	18.8 (3)	43.8 (7)	0.44	12.5 (2)	43.8 (7)	43.8 (7)	0.18
No	33.3 (86)	33.7 (87)	32.9 (85)		34.9 (90)	32.2 (83)	32.9 (85)	
**Age (y)**	36.67 ± 8.44	37.92 ± 8.66	35.61 ± 8.29	0.40	37.70 ± 8.93	36.47 ± 8.30	35.40 ± 8.07	0.18
**Weight (kg)**	79.05 ± 11.56	79.84 ± 8.62	79.13 ± 10.81	0.85	78.70 ± 10.87	79.87 ± 10.07	79.46 ± 10.24	0.74
**BMI (kg/m**^**2)**^	29.91 ± 3.73	30.70 ± 2.98	30.94 ± 3.98	0.13	30.29 ± 3.84	30.72 ± 3.12	30.54 ± 3.84	0.72
**WC (cm)**	97.52 ± 10.13	98.55 ± 7.30	97.91 ± 9.48	0.73	97.36 ± 9.74	98.54 ± 8.41	98.07 ± 8.97	0.67
**WHR (ratio)**	0.93 ± 0.05	0.93 ± 0.04	0.92 ± 0.04	0.39	0.92 ± 0.05	0.93 ± 0.05	0.92 ± 0.04	0.78
**FFM (kg)**	46.72 ± 5.83	46.57 ± 5.06	46.08 ± 5.00	0.69	46.13 ± 5.44	46.41 ± 5.63	46.83 ± 4.83	0.66
**BFM (kg)**	32.31 ± 7.44	32.96 ± 5.87	33.61 ± 8.33	0.48	32.16 ± 7.37	33.48 ± 6.46	33.25 ± 7.96	0.42
**FBS (mg/dl)**	86.62 ± 8.40	87.60 ± 10.32	87.97 ± 10.36	0.66	86.97 ± 10.04	88.86 ± 9.82	86.55 ± 9.13	0.31
**TG (mg/dl)**	121.95 ± 72.62	125.17 ± 71.17	116.88 ± 66.59	0.76	123.62 ± 72.88	119.67 ± 69.43	120.67 ± 68.38	0.93
**Total cholesterol (g/dl)**	176.26 ± 32.79	194.01 ± 40.54	182.59 ± 32.85	**0.007**	183.32 ± 37.58	191.38 ± 39.28	179.15 ± 31.24	0.12
**LDL (mg/dl)**	91.87 ± 23.42	97.10 ± 26.54	94.86 ± 21.94	0.38	96.67 ± 24.75	94.52 ± 26.72	92.28 ± 20.93	0.50
**HDL (mg/dl)**	46.83 ± 11.75	47.67 ± 10.90	45.81 ± 10.04	0.58	47.12 ± 11.51	47.32 ± 11.22	46.02 ± 10.13	0.73
**HOMA index**	3.12 ± 1.17	3.41 ± 1.36	3.51 ± 1.33	0.15	3.43 ± 1.39	3.35 ± 1.22	3.23 ± 1.25	0.61
**hs-CRP (mg/l)**	3.87 ± 4.47	4.21 ± 4.73	4.65 ± 4.78	0.59	4.68 ± 4.87	3.51 ± 4.74	4.32 ± 4.29	0.31
**SBP**	109.37 ± 12.61	113.30 ± 14.33	110.82 ± 13.34	0.14	110.82 ± 12.51	111.03 ± 13.86	111.68 ± 14.21	0.90
**DBP**	75.80 ± 8.42	78.18 ± 9.71	78.77 ± 10.51	0.09	77.40 ± 8.36	77.24 ± 9.93	78.10 ± 10.57	0.82
**PA (met-min/w)**	1032.81 ± 1085.08	960.10 ± 1190.98	937.30 ± 907.63	0.84	919.50 ± 984.98	963.82 ± 1056.73	1054.42 ± 1176.35	0.72

*GL* Glycemic load, *GI* Glycemic index, *BMI* Body mass index, *WC* Waist circumference, *WHR* Waist height ratio, *FFM* Fat free mass, *BFM* Body fat mass, *FBS* Fasting blood sugar, *TG* Triglyceride, *LDL* Low density lipoprotein, *HDL* High density lipoprotein, *hs-CRP* High-sensitivity C-reactive protein, *SBP* Systolic blood pressure, *DBP* Diastolic blood pressure, *PA* Physical activity

^a^Calculated by Chi-square and analysis of variance (*ANOVA*) for qualitative and quantitative variables, respectively

Values are represented as means (SD). Categorical variables: N (%)

### Difference in means of quality-of-life items across two groups of GI and GL

We found decreasing trends for three dimensions of SF-36, including SF-36 –total (*p* = 0.02), RE (*p* = 0.03), and GH (*p* = 0.05) across GL categories, and for role emotional (*p* = 0.03) across GI. But we observed that women in T3 of GL and GI had a significantly higher score of MH compared to T1. Moreover, a significant difference for VT (*p* = 0.03) across GL categories was seen (Table 2).

**Table 2:** Quality of life items across two groups of GI and GL

**Variables**^a^	**GI**					**GL**		
	T1	T2	T3	*P*-value	T1	T2	T3	*P*-value
**SF-36 –TOTAL**	62.30 ± 26.64	63.00 ± 30.52	57.90 ± 30.99	0.56	67.52 ± 26.89	62.14 ± 29.11	53.33 ± 30.79	**0.02**
**General Health**	66.60 ± 15.51	65.59 ± 18.80	67.48 ± 15.67	0.80	64.37 ± 18.19	70.53 ± 13.25	64.38 ± 18.02	**0.05**
**Physical Functioning**	82.83 ± 14.72	80.21 ± 18.09	84.41 ± 14.85	0.31	82.49 ± 16.58	84.30 ± 14.20	80.67 ± 17.27	0.41
**Role Physical**	83.79 ± 36.60	81.34 ± 38.76	82.08 ± 38.63	0.93	80.74 ± 39.23	84.55 ± 35.88	81.42 ± 39.16	0.83
**Role Emotional**	84.31 ± 36.47	80.59 ± 39.84	65.67 ± 47.83	**0.03**	70.07 ± 44.23	86.76 ± 34.13	68.42 ± 46.67	**0.03**
**Social Functioning**	71.16 ± 20.02	72.01 ± 24.66	73.99 ± 24.10	0.77	70.04 ± 23.37	74.08 ± 22.44	72.71 ± 23.59	0.62
**Bodily Pain**	58.07 ± 20.17	61.98 ± 19.89	57.90 ± 20.83	0.43	56.79 ± 20.92	62.79 ± 19.10	58.07 ± 20.75	0.21
**Vitality**	65.94 ± 18.74	68.28 ± 19.31	67.25 ± 17.42	0.78	62.37 ± 19.91	71.69 ± 17.97	66.61 ± 16.82	**0.01**
**Mental Health**	70.17 ± 23.82	79.75 ± 21.58	78.17 ± 21.13	**0.04**	68.19 ± 24.30	81.41 ± 19.93	77.60 ± 21.67	**0.004**
**Health Transition Item**	50.86 ± 26.05	40.67 ± 28.48	47.01 ± 24.81	0.09	44.44 ± 26.45	50.73 ± 28.31	42.50 ± 24.94	0.17

*GL* Glycemic load, *GI* Glycemic index

^a^Calculated by analysis of variance (*ANOVA*)

Values are represented as means (SD)

### Comparison of daily nutrients intake in participants across GI and GL

Selected nutrients and food group intakes of participants across tertiles of GI and GL are presented in Table3. Participants assigned in the highest category of GI were characterized by lower intake of vitamin B12 (*P* = 0.03), vitamin D (*P* = 0.02), and biotin (*P* = 0.02). However, they showed higher intake of vitamin B3 (*P* = 0.04), vitamin B6 (*P* = 0.003), folate (*P* = 0.01), protein (*P* < 0.001), fruits (*P* = 0.02), and tea and coffee (*P* = 0.01). Also, there was a marginally significant difference for intake of fish (*p* = 0.06) and low-fat dairy (*p* = 0.07).

**Table 3:** Energy-adjusted dietary intakes across two groups of GL and GI

**Variables**		**GI**				**GL**		
	**T1**	**T2**	**T3**	***P*** **-value**	**T1**	**T2**	**T3**	***P*** **-value**
Vitamin A (RAE)	809.72 ± 353.09	711.06 ± 347.07	794.62 ± 475.58	0.12	607.02 ± 309.02	776.36 ± 387.25	932.02 ± 444.53	0.50
Vitamin E (mg/day)	16.52 ± 8.38	17.36 ± 10.20	17.72 ± 8.90	0.74	15.62 ± 9.63	17.16 ± 9.89	18.82 ± 7.65	**0.002**
Vitamin B1 (mg/day)	2.02 ± 0.63	1.97 ± 0.57	2.23 ± 0.71	0.53	1.54 ± 0.42	1.99 ± 0.39	2.70 ± 0.51	**0.03**
Vitamin B2 (mg/day)	2.20 ± 0.74	2.07 ± 0.90	2.31 ± 0.76	0.12	1.68 ± 0.55	2.10 ± 0.56	2.79 ± 0.85	0.42
Vitamin B3 (mg/day)	25.29 ± 10.18	23.60 ± 7.92	26.36 ± 8.43	**0.04**	18.73 ± 5.22	24.52 ± 7.80	32.00 ± 8.00	0.80
Vitamin B5 (mg/day)	6.60 ± 2.15	6.20 ± 2.97	6.60 ± 1.94	**0.01**	4.96 ± 1.48	6.20 ± 1.48	8.24 ± 2.71	0.88
Vitamin B6 (mg/day)	2.19 ± 0.75	2.04 ± 0.65	2.22 ± 0.67	**0.003**	1.61 ± 0.43	2.12 ± 0.57	2.72 ± 0.56	0.46
Vitamin B9 (μg/day)	616.97 ± 192.73	660.14 ± 203.31	747.05 ± 233.48	**0.01**	469.14 ± 126.41	641.43 ± 125.07	886.58 ± 177.50	** < 0.001**
VitaminB12 (μg/day)	4.61 ± 2.51	3.82 ± 1.72	4.57 ± 2.79	**0.03**	3.61 ± 1.65	3.99 ± 1.78	5.41 ± 3.11	**0.01**
Vitamin C (mg/day)	188.21 ± 109.22	202.73 ± 152.23	196.18 ± 109.01	0.12	122.21 ± 60.43	196.96 ± 103.33	267.94 ± 148.41	**0.01**
Vitamin D (μg/day)	2.24 ± 1.77	1.75 ± 1.67	1.90 ± 1.40	**0.02**	1.69 ± 1.34	1.85 ± 1.34	2.35 ± 2.05	0.08
Vitamin K (μg/day)	215.44 ± 121.96	188.61 ± 147.79	219.45 ± 260.07	0.59	162.26 ± 100.74	232.58 ± 268.02	228.66 ± 140.63	0.16
Biotin (μg/day)	40.78 ± 14.21	36.22 ± 21.10	38.15 ± 14.29	**0.005**	29.96 ± 11.96	37.94 ± 12.83	47.26 ± 20.08	0.69
Carbohydrate(g/day)	349.27 ± 108.42	362.72 ± 118.58	403.36 ± 126.14	0.15	256.64 ± 55.61	355.36 ± 45.64	503.34 ± 83.56	** < 0.001**
Protein (g/day)	91.45 ± 32.28	80.82 ± 23.32	92.04 ± 26.75	** < 0.001**	68.20 ± 20.72	86.40 ± 23.69	109.71 ± 22.72	**0.02**
Fat (g/day)	90.71 ± 29.24	89.45 ± 33.08	101.50 ± 33.64	0.88	74.28 ± 25.46	92.29 ± 27.76	115.09 ± 30.09	** < 0.001**
Fiber(g/day)	42.16 ± 15.92	44.34 ± 19.30	49.01 ± 20.69	0.59	30.90 ± 11.24	45.25 ± 13.57	59.36 ± 18.94	**0.002**
**Food groups**								
Whole grains	7.60 ± 9.41	8.21 ± 12.40	6.77 ± 8.75	0.39	5.79 ± 8.21	7.74 ± 10.51	9.04 ± 11.69	0.61
Refined grains	438.25 ± 215.60	465.65 ± 246.88	509.03 ± 228.84	0.64	353.19 ± 179.59	422.56 ± 133.97	637.18 ± 260.49	**0.01**
Red meat	20.95 ± 17.92	20.22 ± 18.89	22.54 ± 17.03	0.78	14.82 ± 11.56	20.40 ± 19.73	28.49 ± 18.71	0.72
Fish	13.41 ± 16.17	9.52 ± 8.09	10.99 ± 10.99	**0.06**	10.25 ± 9.25	11.39 ± 13.29	12.29 ± 13.82	0.17
Fruits	505.52 ± 320.84	568.37 ± 386.66	524.32 ± 303.42	**0.02**	323.15 ± 194.00	525.87 ± 272.53	749.18 ± 378.00	**0.004**
Vegetables	456.16 ± 234.93	417.24 ± 287.34	424.15 ± 256.71	0.19	336.97 ± 205.65	458.34 ± 262.59	502.24 ± 280.12	0.22
Low fat dairy	318.15 ± 237.11	257.54 ± 220.65	278.40 ± 210.83	**0.07**	258.28 ± 180.34	257.76 ± 182.86	338.06 ± 284.59	0.14
High fat dairy	100.70 ± 138.71	104.80 ± 149.33	121.80 ± 137.03	0.97	67.98 ± 111.98	105.48 ± 122.29	153.44 ± 171.11	0.41
eggs	21.34 ± 12.20	19.71 ± 13.03	24.09 ± 17.28	0.33	16.55 ± 9.64	23.29 ± 13.65	25.30 ± 17.49	**0.04**
legumes	57.99 ± 46.02	50.50 ± 41.09	48.52 ± 35.56	0.10	48.66 ± 43.85	52.03 ± 35.80	56.32 ± 43.44	0.20
Nuts	14.26 ± 11.95	13.86 ± 18.60	15.74 ± 18.22	0.83	9.03 ± 8.52	13.26 ± 16.68	21.57 ± 19.74	0.89
Tea & coffee	580.08 ± 467.25	689.78 ± 552.76	949.38 ± 1081.78	**0.01**	559.16 ± 448.66	709.86 ± 1066.84	950.23 ± 590.53	**0.05**
Sugar sweetened beverages	18.24 ± 50.65	20.30 ± 38.90	29.08 ± 66.54	0.71	8.77 ± 13.57	25.37 ± 66.26	33.48 ± 60.66	0.55
Fast food	25.43 ± 27.49	21.35 ± 17.56	26.35 ± 33.73	0.55	20.01 ± 20.02	21.27 ± 23.58	31.85 ± 34.26	0.22

All the variables adjusted for energy intake

*GL* Glycemic load, *GI* Glycemic index

^†^Calculated by multivariate analysis of covariance (ANCOVA)

Values are represented as means (SD)

The results showed that intake of vitamin E (*P* = 0.002), vitamin B1(*P* = 0.03), vitamin B9 (*P* < 0.001), vitamin B12 (*P* = 0.01), vitamin C (*P* = 0.01), carbohydrate (*P* < 0.001), protein (*P* = 0.02), fat (*P* < 0.001), fiber (*P* = 0.002), refined grain (*P* = 0.01), fruits (*P* = 0.004), eggs (*P* = 0.04), and tea and coffee (*P* = 0.05) increased significantly across tertiles of GL (Table 3).

### Association of the GL and GI with quality of life

A significant negative association between the GL and quality of life (β = -0.08, 95%CI = -0.14 -0.03, *p* = 0.002) was seen in the crude model. Moreover, this significant negative association was maintained after adjusting for confounding factors, such as age, PA, BMI, education, job, smoking, and marriage (β = -0.07, 95%CI = -0.13 -0.01, *p* = 0.01). However, no significant association of the GI with QoL was seen in the crude or adjusted models (β = -0.03, 95%CI = -0.81- 0.75, *p* = 0.93) (Table 4).

**Table 4:** Association of GI and GL with quality-of-life among obese and overweight female subjects

		**SF-36 –TOTAL**^**a**^		
		**Β**	**95 CI**	***P*** **-value**
**GI**	Crude	-0.07	-0.79 to 0.63	0.83
	*M1*	0.03	-0.74 to 0.80	0.93
	*M2*	-0.03	-0.81 to 0.75	0.93
**GL**	Crude	-0.08	-0.14 to -0.03	**0.002**
	*M1*	-0.07	-0.13 to -0.01	**0.01**
	*M2*	-0.07	-0.13 to -0.01	**0.01**

M1: Adjusted for age, PA, BMI

M2:Adjusted for age, PA, BMI, education, job, smoking, marri

^a^Linear regression; CI: confidence interval; GL: glycemic load; GI: glycemic index

## Discussion

In this article, we examined the association between GI/GL of carbohydrate and QoL in overweight and obese women. In summary, we found that higher GI diet was positively associated with total cholesterol. We found that higher GI diet can lower RE and QoL total score across GL categories. Moreover, we found a marginal decreasing trend for GH across GL category. In our study, women in T3 of GL and GI had a significantly higher score of MH compared to T1, in addition, a significant difference was found for vitality across GL categories. Participants assigned in the highest category of GI showed lower intake of vitamin B12, vitamin D, and biotin, and higher intake of vitamin B3, vitamin B6, folate, protein, fruits, and tea and coffee. Additionally, a borderline significant difference for intake of fish and low-fat dairy was observed. The results indicated that intake of vitamin E, vitamin B1, vitamin B9, vitamin B12, vitamin C, carbohydrate, protein, fat, fiber, refined grain, fruits, eggs, and tea and coffee increased significantly across tertiles of GL, and we did not find any significant association about GI and GL with other components of QoL. Nutrition, holistically, may be associated with various aspects of QoL, however, as carbohydrate is, typically, the main component of calories consumed, we examined how GI and GL of carbohydrate can affect QoL [29, 30]. After examining for general characteristics of participants across two groups of GI and GL, we found that higher GI diet can induce increased total cholesterol. While some studies support the hypothesis that a high-GI diet unfavorably increases the risk dyslipidemia and cardio metabolic disorders [7, 8], some other studies observed the opposite association [9, 10]. Indeed, this association can be explained by elevated level of insulin concentration just after high GI (HGI) meal consumption and hypoglycemia which appears after 4–6 h. Elevated insulin, glucose, and free fatty acid level, after HGI meal consumption, can induce IR which can, in turn, cause dyslipidemia [11]. Our results showed that RE decreased across GI categories, in addition to RE, GH and QoL total score decreased across GL categories. This result was in line with previous studies in women with poly-cystic ovary syndrome, women's hormonal changes in emotional situation such as excessive happiness or sadness, impact their eating habits, they tend to eat more comfortable and energy dense food that are rich in sugar with higher GI and GL to lower their RE [31–33].We found GH, another component of QoL, was marginally associated with increasing trend of GL categories. GH is rated based on one's self-perception of his or her health status and depends on many factors, such as having mental and physical disorders and chronic disease [34]. As reported in previous studies, lower GL diet may lead to important reductions in blood pressure [35]. Additionally, low-GI and -GL diets tend to consist of low total calories, and this may promote energy intake regulation, which often leads to weight loss, and has been identified as a strong predictor of lower blood pressure [35]. We found that women in T3 of GL and GI had a significantly higher score of MH compared to T1. Although some studies reported protective effects of low GI diet in relation to MH, some other studies support our results where higher GL and GI diets were associated with lower prevalence mental disorders, depression, and psychological distress [5], with studies emphasizing the role of serotonin as a mediator of mood pathways. HGL diets increase insulin secretion, which can increase the proportion of tryptophan circulation to large neutral amino acids (LNAAs), and even promote transportation of tryptophan across the blood–brain barrier to predict brain serotonin synthesize [36]. Most studies have reported that HGL diets are accompanied with fatigue and have adverse effects on vitality, while our findings pertaining to vitality were different [37], which could be explained by insulin secretion and serotonin synthesis induced by HGL diet that can increase vitality [15]. HGL diets, compared to isoenergetic low glycemic load (LGL) diets, can increase glycogen storage within muscle and liver [38]. Indeed, higher glycogen may be related to feelings of being cheerful and postpone premature fatigue, which can increase vitality [39, 40]. Participants with HGI carbohydrate consumption, in contrast with participants in low GI categories, had lower intake of vitamin B12, vitamin D, and biotin, where these nutrients have been reported to positively effect the nervous system, and neurotransmitter transportation and synthesis [12–14]. Studies have shown that increases in the intake of vitamin B12 led to an improvement in QOL measures, which can be referred to emotional well-being [41]. Therefore the role of B12 in the RE can justify our results; across the GI categories, the RE has decreasing trends. We observed a significant negative association between QoL and GL, but not GI, among overweight and obese women, even after adjustment for confounders. Most studies support the notion that HGL diets can cause lower QoL by affecting mental and physical dysfunction, and inducing improper body shape and chronic disorders [5, 7, 15].

Our study has some limitations that should be noted. First, due to the cross-sectional design of this study, underlying causative factors cannot not be inferred. Second, this study was conducted on women only, which reduces the generalizability of the study results. Third, the sample size was not large enough to detect some relationships in this cross-sectional study. Fourth, FFQ and QoL questionnaires were self-report measures, which are susceptible to misreporting. Nevertheless, the strengths of the study were that all components of QoL were assessed, while other studies just examined one or two components in relation to carbohydrate quality. In addition, we used FFQ questionnaires specifically validated in the Iranian population. Another strength is that previous studies assessed the effect of carbohydrate quality with different components of QoL separately, while this original article aimed to evaluate the relationship between GI, GL, and all component of QoL in overweight and obese women, for the first time.

## Conclusion

Based on our findings, a significant negative association was observed between QoL and GL, but not GI, among overweight and obese women. This finding highlights the importance of proper nutrition for QoL. However, more studies needed, particularly with cohort and RCT designs, to ameliorate the limitations of the present study.

## Data Availability

The data that support the findings of this study are available from correspond author but restrictions apply to the availability of these data, which were used under license for the current study, and so are not publicly available. Data are however available from the authors upon reasonable request and with permission of correspond author.

## Abbreviations

BMI: Body mass index
BP: Bodily pain
FBS: Fasting blood glucose
FFM: Fat free mass
FM: Fat mass
GH: General health
GI: Glycemic index
GL: Glycemic load
GR: Glycemic response
hs-CRP: High-sensitive C-reactive protein
FFQ: Food frequency
HGL: High glycemic load
HOMA: Homeostasis model assessment
HDL: High-density lipoprotein
IPAQ: International physical activity questionnaire
LGL: Low glycemic load
LDL: Low-density-lipoprotein
MH: Mental health
PA: Physical activity
PF: Physical functioning
QoL: Quality of life
RE: Role emotional
RP: Role-physical
SF: Social functioning
SMM: Skeletal muscle mass
TC: Total cholesterol
TG: Triglyceride
VT: Vitality
WC: Waist circumference
WHR: Waist to hip ratio
WHO: World health organization
